# Profiles of Parents’ Beliefs About Their Child’s Intelligence and Self-Regulation: A Latent Profile Analysis

**DOI:** 10.3389/fpsyg.2020.610262

**Published:** 2020-12-09

**Authors:** Maren Stern, Silke Hertel

**Affiliations:** Institute of Education Science, Heidelberg University, Heidelberg, Germany

**Keywords:** implicit theories, intelligence, self-regulation, parents, latent profile analysis

## Abstract

This study examined parents’ implicit theories of intelligence and self-regulation from a person-centered perspective using latent profile analysis. First, we explored whether different belief profiles exist. Second, we examined if the emergent belief profiles (1) differ by demographic variables (e.g., age, education, child’s self-regulation) and (2) are related to parents’ failure beliefs, goal orientation (i.e., learning goals, performance-approach goals, performance-avoidance goals), and co-regulatory strategies (i.e., mastery-oriented and helpless-oriented strategies). Data were collected from *N* = 137 parents of preschoolers who answered an online survey comprising their implicit theories about the malleability and relevance of the domains (a) intelligence and (b) self-regulation. We identified three belief profiles: profile 1 (9% of the sample) displayed an entity theory, profile 2 (61% of the sample) showed a balanced pattern of both domains of implicit theories, and profile 3 (30% of the sample) was characterized by high incremental self-regulation theories. Analyses showed that parents differed significantly in education and their perception of child self-regulatory competence depending on profile membership, with parents in profile 1 having the lowest scores compared to parents of the other profiles. Differences in parents’ failure beliefs, goal orientation, and co-regulatory strategies were also found depending on profile membership. Parents in profile 3 reported failure-is-enhancing mindsets, and mastery-oriented strategies significantly more often than parents in profiles 1 and 2. The results provide new insights into the interplay of important domains of implicit theories, and their associations with parents’ failure beliefs, goal orientation, and co-regulatory strategies.

## Introduction

Many parents have concrete beliefs about their children’s abilities. For example, parents may view their children’s abilities as malleable and changeable by effort or rather believe that their children have innate competencies that are relatively fixed and cannot be changed. Parents’ cognitions have important short- and long-term effects on parenting practices and child development (Bornstein et al., 2018). More precisely, parents’ implicit theories influence parents’ goal orientation, their co-regulatory strategies, and consequently their child’s self-regulation (Ames and Archer, 1987; Grolnick et al., 2002; Pomerantz and Dong, 2006; Blackwell et al., 2007; Moorman and Pomerantz, 2010; Burnette et al., 2013; Jiang et al., 2019).

Although the importance of implicit theories is evident, relatively little is known about how different domains (e.g., intelligence, self-regulation) and dimensions (e.g., malleability, relevance) of implicit theories co-occur in everyday situations affecting parents’ attitudes (e.g., failure beliefs, goal orientation) and co-regulatory strategies. This lack of attention to interaction processes of different domains is surprising, given that individuals can hold different implicit theories in different domains and attributes at the same time (Dweck et al., 1995; Tabernero and Wood, 1999; Muenks et al., 2015; Haimovitz and Dweck, 2017). For example, some parents may view their children’s ability in one domain (e.g., self-regulation) to be malleable while considering their children’s ability in another domain (e.g., intelligence) to be relatively fixed. Other parents may think that both domains of abilities are malleable but that only one of these is relevant for their children’s success. To date, research on implicit theories has predominantly focused on implicit theories of intelligence (Dweck, 2000; Moorman and Pomerantz, 2010) while ignoring the domain of self-regulation. Since parents play an important role in children’s self-regulatory development, parents’ implicit theories of self-regulation should play an important role in predicting self-regulatory processes.

Therefore, this study examined how implicit theories co-occur within parents using latent profile analysis (LPA). LPA is a person-centered approach that aims to identify unobserved subgroups based on the similarity of the sample on observed variables (Collins and Lanza, 2009). The variables used for the LPA comprised two domains of children’s abilities: *intelligence* and *self-regulation*, each including two dimensions: *malleability* and *relevance for success*. We then analyzed how the emergent belief profiles are composed with respect to demographic variables. Finally, we explored how different belief profiles relate to parents’ attitudes (i.e., failure beliefs, goal orientation) and co-regulatory strategies.

## Theoretical Background

### Implicit Theories of Abilities

Implicit theories are belief systems about human attributes and abilities that help individuals to explain and understand their world (Lüftenegger and Chen, 2017). There is a long tradition in research following Carol Dweck’s social cognitive theory (Dweck and Leggett, 1988) examining the malleability of abilities. She distinguishes two types of implicit theories: incremental theories and entity theories. *Incremental theories* refer to viewing abilities as malleable and changeable by effort while *entity theories* refer to viewing abilities as innate competencies that are rather fixed. So far, these implicit theories were mainly examined in children and students, showing that incremental theories are related to higher motivation, persistence, adaptive learning strategies, and academic achievement (Dweck and Leggett, 1988; Blackwell et al., 2007).

Although there is a wealth of evidence that implicit theories are relevant determinants of motivation, cognition, and behavior in learning and achievement settings (Blackwell et al., 2007; Burnette et al., 2013), parental implicit theories have gained attention only recently. Parental implicit theories refer to beliefs parents have about the abilities of their children. These can refer to an array of abilities and domains such as intelligence (Dweck, 2000; Pomerantz and Dong, 2006), math and verbal ability (Muenks et al., 2015), or failure (Haimovitz and Dweck, 2016). These implicit theories from various domains can correlate but findings suggest relatively independent constructs (Dweck et al., 1995; Tabernero and Wood, 1999; Haimovitz and Dweck, 2016). This means that individuals can hold an incremental theory in one domain but an entity theory in another domain (Schroder et al., 2016).

In the context of parents, past research has primarily focused on parents’ implicit theories of *intelligence* (Dweck, 2000; Pomerantz and Dong, 2006; Moorman and Pomerantz, 2010; Rautiainen et al., 2016). The interest in the domain of intelligence originates from broad evidence suggesting that implicit theories of intelligence have important effects on academic and emotional functioning (for a meta-analytic review see Costa and Faria, 2018). Inspired by research about children’s implicit theories of intelligence, researchers have asked if parents’ implicit theories are also consequential for children’s implicit theories as well as parents’ learning and achievement-related behaviors (e.g., Rautiainen et al., 2016) as parents’ play an important role in children’s socialization (Taylor et al., 2004). Initial studies indicate that parents’ incremental theories predict children’s outcomes (e.g., children’s incremental theories, achievement) and parental learning-related behaviors (Pomerantz and Dong, 2006; Moorman and Pomerantz, 2010; Muenks et al., 2015; Matthes and Stoeger, 2018).

In children’s development, intelligence is not the only significant domain that influences parents’ and their children’s beliefs and in turn the associated consequences. The concept of *self-regulation* receives high attention in both scientific and popular scientific literature and is known as a central construct of psychology (Vohs and Baumeister, 2013). Self-regulation is defined as the ability “to regulate affect, attention, and behavior to respond effectively to both internal and environmental demands” (Raffaelli et al., 2005, p. 54). Self-regulation develops in early childhood and predicts a range of social-emotional, health-related, and academic outcomes (Moffitt et al., 2011; McClelland and Cameron, 2012; Neuenschwander et al., 2012; Valiente et al., 2013). However, what individuals believe about the malleability and relevance of self-regulation remains largely unexplored. Initial studies indicate that these implicit theories of self-regulation are associated with self-regulatory processes such as goal orientation and learning strategy use (Hertel and Karlen, under review; Stern et al., under review), and influence effort and perseverance (Mrazek et al., 2018).

However, research suggests that it is not only the question of whether parents believe that abilities are malleable (Stern et al., under review); another important dimension of implicit theories is the question of the abilities’ *relevance for success* (Spinath, 2001). Individuals can hold different opinions about how relevant abilities are for the success in particular tasks (e.g., the relevance of intelligence for school achievement; Schlangen and Stiensmeier-Pelster, 1997; Spinath and Schöne, 2003). Inspired by Wigfield and Eccles (2000) expectancy-value theory of motivation it can be assumed that the belief about the relevance of a certain ability is an important predecessor of motivation and influences behavior. For example, if parents believe that a certain ability is a relevant variable for their children’s success in a specific context, they will promote and support their children’s development. These beliefs, in turn, may affect the relation between implicit theories about the malleability of abilities and behavior: Only if individuals believe that a certain ability is a relevant variable individuals’ incremental or entity theories may become effective (Spinath and Schöne, 2003). Malleability and relevance for success seem to be moderately correlated dimensions of implicit theories that both have beneficial effects explaining links between implicit theories and learning-related outcomes (Hertel and Karlen, under review; Stern et al., under review). However, a simultaneous consideration of both dimensions is rare in the context of research concerning parents’ implicit theories.

### Implicit Theories and Failure Beliefs

Implicit theories are most powerful in challenging and demanding situations (Dweck and Leggett, 1988; Blackwell et al., 2007). Dweck and Leggett (1988) argue that implicit theories are related to the attribution of failure and individuals’ behaviors: Individuals with an incremental theory attribute failure to a lack of effort. Incremental theorists are more likely to persist through failure as they see failure as an opportunity for learning. In contrast, individuals with an entity theory attribute failure to a lack of ability. Entity theorists tend to give up in the face of failure because they see failure as a sign of being incompetent (Dweck and Leggett, 1988; Blackwell et al., 2007; King, 2017).

In the context of parents, failure beliefs are of special interest. Especially during early childhood, children are still developing their skills and are often in the face of failure. Here, parents play an important role to support their children and enable them to solve challenging tasks (Bernier et al., 2010). Haimovitz and Dweck (2016) have identified two different failure beliefs of parents: a failure-is-enhancing mindset and a failure-is-debilitating mindset. Parents with a *failure-is-enhancing mindset* view failure as “an enhancing experience that facilitates learning and growth […, while parents with a *failure-is-debilitating mindset* believe] that failure is a debilitating experience that inhibits learning and productivity” (Haimovitz and Dweck, 2016, p. 860). Empirically, these beliefs relate to parenting practices and children’s intelligence theories: Parents, who view failure as debilitating show more performance-oriented responses, report less support for their children‘s learning, and more concerns about their children’s performance and lack of ability compared to parents with a failure-is-enhancing mindset (Haimovitz and Dweck, 2016). Moreover, parents with a failure-is-debilitating mindset have children who believe that intelligence is fixed. However, the link between parents’ failure beliefs and parents’ implicit theories is not well-understood so far. There is some evidence that parents’ implicit theories and failure beliefs are independent constructs, whereas there is also some suggestion that parents’ entity theories are positively correlated to their failure-is-debilitating mindsets (see Haimovitz and Dweck, 2016). The question also arises if the relation between implicit theories and failure beliefs is domain-specific. More specifically, some parents, for example, may believe that failure is debilitating to develop self-regulatory abilities but enhancing to increase intelligence. Therefore, it seems important to examine these mechanisms in more detail and take further domains and dimensions of implicit theories into account (e.g., implicit theories of self-regulation) to better understand how parents’ implicit theories and failure beliefs are related.

### Implicit Theories and Goal Orientation

Implicit theories are significantly linked to goal orientation (Burnette et al., 2013): Individuals perceiving abilities as malleable pursue *learning goals* to increase their skills, while individuals holding an entity theory pursue performance goals to secure positive judgments (*performance-approach goal orientation*) or avoid challenging tasks to prevent negative judgments (*performance-avoidance goal orientation*) (Dweck, 1986). Applied to parenting, parents with learning goals want their child to develop skills, whereas parents with performance goals want to demonstrate their children’s competences (performance-approach) or avoid situations where their child might perform worse than others (performance-avoidance) (Mageau et al., 2016). Parental goal orientation affects parents’ co-regulatory strategies (e.g., autonomy support, control; Gonida and Cortina, 2014; Mageau et al., 2016) as well as children’s beliefs, motivation, and performance (Gottfried et al., 1994; Grolnick et al., 2002; Gunderson et al., 2013). For example, parents with performance goals provide more controlling behavior to their children compared to parents with learning goals (Grolnick et al., 2002). While performance-avoidance goals have proved predominantly maladaptive (e.g., poor performances, test anxiety, low help-seeking behavior; for a review see Moller and Elliot, 2006), performance-approach goals can have both positive and negative effects (Mageau et al., 2016).

Meta-analytical findings by Burnette et al. (2013) with 113 studies across diverse contexts and populations suggest positive associations between incremental theories and learning goals as well as between entity theories and performance-avoidance goals. No substantial relation for performance-approach goals was found. In contrast, in the specific context of parents, the effect of learning goals but not of performance-avoidance goals could be confirmed (Stern et al., under review). One explanation might be that parents’ performance-avoidance goals were low overall. Moreover, parents’ implicit theories about the relevance of abilities might play an important role, as these have been found to be positively correlated with parents’ performance-approach goals (Stern et al., under review). Previous research has especially used incremental theories of intelligence to predict goal orientation and ignored implicit theories about the relevance of abilities. A simultaneous consideration of two domains of implicit theories about the malleability *and* relevance of abilities might explain the complex pattern of associations between parents’ implicit theories and goal orientation.

### Implicit Theories and Co-regulatory Strategies

Parents’ co-regulatory strategies, in the sense of attempts to modify children’s thoughts, emotions, and behavior (Colman et al., 2006; Pauen, 2016), are especially relevant in early childhood when self-regulatory abilities are developing and children are still dependent on their parents’ support (Kopp, 1982; Bernier et al., 2010; Valcan et al., 2018). While *mastery-oriented co-regulatory strategies* (e.g., warmth, inductive discipline, scaffolding, autonomy support) are associated with higher self-regulatory abilities, *helpless-oriented co-regulatory strategies* (e.g, control, intrusiveness) are related to lower self-regulatory abilities of children.

Research across different domains and populations has shown that a person’s implicit theory predicts mastery- and helpless-oriented strategies (Burnette et al., 2013). Applied to parenting, one may assume that parents with incremental theories are more likely to use mastery-oriented strategies that help their child to learn (e.g., remaining encouraging; holding discussions; calling for self-regulation) because the child’s abilities reflect learning processes that can be promoted. In contrast, entity theorists may tend to employ helpless-oriented strategies (e.g., using negative pressure for example by forcing the child to comply; giving in) as a reaction of poor performances that reflect stable abilities and consequently permanent deficits. This line of reasoning is substantiated by evidence that parents’ implicit theories are important determinants of parents’ co-regulatory strategies: Parents who believe that abilities (e.g., intelligence, math, and verbal abilities) are stable show more controlling and performance-oriented behaviors than parents with incremental theories (Moorman and Pomerantz, 2010; Muenks et al., 2015). Nevertheless, it is unclear whether the effects are stronger for some parents than others because past studies used experimental manipulations (Moorman and Pomerantz, 2010) or measured limited demographic characteristics (Muenks et al., 2015). Using a person-centered approach and examining belief profiles and their relations to parents’ co-regulatory strategies could help close this research gap.

### Sociodemographic Group Differences in Implicit Theories of Abilities

Regarding sociodemographic variables that shape parents’ implicit theories, empirical investigations are rare. Increasing research examines group differences in implicit theories by demographic variables such as gender, age, and educational level. However, it is still under debate if and how demographic variables are and should be related to implicit theories. Gender is mostly unrelated to implicit theories (Pomerantz and Dong, 2006; Burnette et al., 2013; Muenks et al., 2015; Jiang et al., 2019). Anyhow, parents’ gender may shape parents’ implicit theories, as mothers’ and fathers’ values and understanding of their children’s upbringing may disagree (e.g., Lareau, 2000). Parents’ implicit theories may also differ by their children’s gender: Parents are more prone to attribute boys’ achievement to talent and girls’ achievement to effort (e.g., Eccles et al., 1990). Furthermore, some researchers argue that girls (especially high-achieving girls) have a lower tendency for new and difficult tasks and attribute failure to a lack of ability (i.e., holding entity theories), compared to boys who tend to hold incremental theories (Dweck, 1986; Chen, 2012; Diseth et al., 2014). Concerning age differences, some studies report that young students tend to overestimate their skills (Hasselhorn, 2005) and therefore hold incremental theories more likely (Chen, 2012). Given that beliefs stabilize with age, no age differences are expected for adults (Pomerantz and Dong, 2006; Jiang et al., 2019). Regarding parents’ educational level, some studies point out that parents’ incremental theories are linked to a higher level of education (Pomerantz and Dong, 2006; Muenks et al., 2015; Jiang et al., 2019). Other researchers (Rautiainen et al., 2016) argue that parents with an academic education tend to hold an entity theory because they support the theory of natural giftedness (Räty and Snellman, 1998) but could not support this hypothesis empirically. Finally, the question arises on how parents’ perceptions of their children’s competence affect parents’ implicit theories. Haimovitz and Dweck (2017) have found that parents’ perceptions of their children’s competence are partly related to parents’ implicit theories. Research from extended literature shows that implicit theories of intelligence are largely unrelated to one’s actual personality and intelligence (Spinath et al., 2003). Overall, these results represent high inconsistency and more studies are needed to illuminate the contribution of person-specific characteristics.

### A Person-Centered Approach to Implicit Theories

The current study uses a person-centered approach by studying patterns of implicit theories in parents. Whereas *variable-centered approaches* (e.g., regressions, path analysis) examine relationships among variables on average, *person-centered approaches* describe relationships among persons by identifying subpopulations depending on their scores on multiple variables of interest (Lubke and Muthén, 2005). The latent profile analysis (LPA) is one of the person-centered approaches and offers several advantages. First, the number of profiles result from empirical fit indices that specify the optimal number and the researcher does not have to determine a number a priori. Second, individuals are not assigned to a specific profile absolutely, but each individual’s probability of memberships for each profile are calculated. LPA is particularly suitable for exploratory research questions and is increasingly used in research on beliefs and attitudes, for example, students’ implicit theories and epistemic beliefs (Chen, 2012; Hertel et al., 2019), or parents’ self-efficacy beliefs (Junttila and Vauras, 2014). This method is particularly useful in this field of research, as individuals may hold different beliefs and attitudes in various domains simultaneously, which results in different configurations of beliefs. Using a variable-centered method might conceal important results and implications. To our knowledge, no study has used LPA to examine implicit theories of abilities in parents so far.

We assume that implicit theories about the malleability and relevance of different domains may co-occur within persons. The present study aims to explore those individual belief profiles that naturally arise among parents of preschoolers. As already described, some parents may hold incremental theories (or entity theories) in different domains at the same time, whereas other parents may hold incremental theories in one domain but entity theories in the other domain, for example. Thus, we examine whether different profiles of implicit theories of intelligence and self-regulation exist. Moreover, we argue that different profiles are differentially adaptive or maladaptive concerning parents’ attitudes (i.e., failure beliefs, goal orientation) and co-regulatory strategies (i.e., mastery- and helpless-oriented strategies). Past research using a variable-centered method shows that parents’ incremental theories are beneficial to learning goals and co-regulatory strategies while entity theories enhance performance-oriented behaviors and children’s helplessness (Moorman and Pomerantz, 2010; Muenks et al., 2015; Jiang et al., 2019). However, when incremental and entity theories co-occur within different domains, the positive effects of incremental theories in one domain might be less strong when parents hold entity theories in another domain. Similarly, incremental theories in one domain might partly counteract the effects of entity theories in the other domain. Therefore, we examine which of the emergent belief profiles are most adaptive for parents’ attitudes and behavior. More precisely, three different research questions guided the present study:

(1) What different belief profiles emerge from measures of parents’ implicit theories of intelligence and implicit theories of self-regulation?
(2) How do these emergent belief profiles differ by parents’ and children’s demographic variables?
(3) How do these emergent belief profiles relate to parents’ failure beliefs, goal orientation, and co-regulatory strategies?

## Materials and Methods

### Participants

Two hundred and fifty-four persons were recruited for an online survey study by social-network-platforms and announcements in kindergartens in southwest Germany. The study was created with the online tool Soscisurvey (Leiner, 2019) and distributed via https://www.soscisurvey.de. As an incentive, participants were offered attractive lottery prizes (six vouchers worth 50–150 Euro). For the present study, we recruited parents of children aged three to six years. One hundred and fifty-two persons finished the questionnaire, leading to a dropout rate of 40% that is slightly higher than the reported average rate of 34% for online studies (Musch and Reips, 2000). The increased dropout rate might be due to technical problems when filling out the questionnaire on smartphones. Fifteen persons were excluded from the analysis because of implausible response patterns, distractions, or not complying with the inclusion criteria (child’s age: 3–6 years), leading to a final sample of 137 parents (87% mothers). Parents’ mean age was 37.42 years (*SD* = 4.85) and they had at least one child (75%). The majority of parents had at least a higher technical college qualification (79%), worked part-time (80%), and were not single parents (95%). Parents were asked to refer to their child aged three to six years when filling out the questionnaire; the mean age of the child was 4.65 (*SD* = 1.08); 55% of the parents thought about their daughter.

### Measures

#### Implicit Theories of Self-Regulation

We used the recently modified and validated *Parents’ Implicit Theories of Self-Regulation scale* (PITSR, Stern et al., under review), assessing parents’ malleability and relevance theories of self-regulation. The two dimensions were assessed by three items, using a five-point-scale adapted to the item content: *malleability* of their child’s self-regulation (e.g., “My child has a certain ability to self-regulate and this … cannot be changed/can be changed,” α = 0.75) and *relevance* of their child’s self-regulation for success (e.g., “Good performance of my child… does not require competencies in self-regulation/does require competencies in self-regulation,” α = 0.73). Higher values indicated more agreement of an incremental theory and higher relevance of self-regulation for success.

#### Implicit Theories of Intelligence

We used modified scales assessing parents‘ implicit theories of intelligence (“Skalen zur Erfassung subjektiver Überzeugungen zu Bedingungen von Erfolg in Lern- und Leistungskontexten,” SE-SÜBELLKO-ST, Spinath and Schöne, 2003; Stern et al., under review). Two dimensions were assessed by three items that could be answered using a five-point-scale adapted to the item content: *malleability* of their child’s intelligence (e.g., “My child possesses a certain amount of intelligence and this … cannot be changed/can be changed,” α = 0.90) and *relevance* of their child’s intelligence for success (e.g., “Good performance of my child… does not require a lot of intelligence/does require a high amount of intelligence,” α = 0.71). Higher values indicated more agreement of an incremental theory and higher relevance of intelligence for success.

#### Failure Beliefs

We used scales assessing parents’ failure beliefs (Haimovitz and Dweck, 2016), translated and adapted them by referring specifically to their child’s failure experiences. Three items described a failure-is-enhancing mindset (e.g., “Experiencing failure facilitates my child’s learning and growth,” α = 0.82) and three items described a failure-is-debilitating mindset (e.g., “Experiencing failure debilitates my child’s learning and growth,” α = 0.77). All items were rated on a scale ranging from *extremely untrue* (1) to *extremely true* (5). Items of the failure-is-debilitating mindset were reverse-scored and averaged with all items to a composite score. Thus, higher numbers indicated a more enhancing view of failure.

#### Goal Orientation

We used scales assessing parents’ goal orientation (“Skalen zur Erfassung der Lern- und Leistungsmotivation“-Questionnaire, SELLMO, Spinath and Schöne, 2019) and adapted them for parents of preschoolers by removing school references. Three dimensions of goal orientation were assessed by eight items each: *learning goals* (e.g., “It is important to me that my child acquires a deep understanding of the content,” α = 0.69), *performance-approach goals* (e.g., “It is important to me that my child shows that s/he masters the contents,” α = 0.84) and *performance-avoidance goals* (e.g., “It is important to me that nobody notices when my child does not understand the content,” α = 0.83). All items were rated on a scale ranging from *totally disagree* (1) to *totally agree* (5).

#### Co-regulatory Strategies

We used the revised version of the IMpulse-MAnagement from Infancy to Preschool questionnaire (IMMA 1–6; Pauen et al., 2019) for assessing parents’ responses to their child’s behavior. M*astery-oriented strategies* were assessed with four items of the dimension *praising* (e.g., “I praise her/him explicitly when s/he does what I desire,” α = 0.84), five items of the dimension *negotiating/discussing* (e.g., “I negotiate a solution with the child when s/he does not do what I desire,” α = 0.75), four items of the dimension *distraction* (e.g., “I try to distract her/him when s/he is frustrated because of not achieving what s/he has planned,” α = 0.84), and three items of the dimension *call for self-regulation* (e.g., “I tell her/him not to get upset when s/he is frustrated because of not achieving what s/he has planned,” α = 0.71). One item of *call for self-regulation* was excluded due to poor internal consistency. *Helpless-oriented strategies* were assessed with four items of the dimension *giving in* (e.g., “I give up when s/he does not do what I desire,” α = 0.89), and eleven items of the dimension *negative pressure* (e.g., “I force the child to comply when s/he does not do what I desire,” α = 0.89). All items were rated on a scale ranging from *never* (1) to *always* (6).

#### Child’s Self-Regulation

Parents’ perception of their child’s self-regulatory competence was assessed with the subscale *Effortful Control* of the German very short form of the Children’s Behavior Questionnaire (CBQ; Putnam and Rothbart, 2006). Parents reported their child’s reaction or behavior in the past six months in different situations on twelve items (e.g., “Is good at following instructions,” α = 0.68) on a scale ranging from *extremely untrue* (1) to *extremely true* (7).

### Analysis

Belief profiles were created through Latent Profile Analysis using Mplus 7.31 (Muthén and Muthén, 2014). Latent Profile Analysis identifies latent homogenous groups (profiles) of individuals that have similar values on the clustering variables (latent profile indicators) by using probabilistic models of subgroup membership (Vermunt and Magidson, 2004). In the present study, four latent profile indicators were used: incremental theory of intelligence, relevance theory of intelligence for success, incremental theory of self-regulation, and relevance theory of self-regulation for success.

Model fit statistics were calculated to identify the number of profiles (Geiser, 2010; Williams and Kibowski, 2016), including Entropy values, Akaike’s Information Criterion (AIC), Bayesian Information Criterion (BIC), and sample size adjusted BlC (aBIC) with higher Entropy values and lower AIC, BIC, aBIC indicating better fit. Lo-Mendell-Rubin (LMR), where *k* and *k*–1 number of profiles were compared, was also conducted. Furthermore, the characteristics of each profile (e.g., size) and interpretability were also considered in the final solution.

In order to explore how the emergent belief profiles differ by demographic variables, parents’ goal orientation, failure beliefs, and co-regulatory strategies (see research questions two and three), Mplus’ auxiliary (BCH) function was employed. The BCH method uses a weighted multiple group analysis and estimates the association between the categorical latent variable and the dependent continuous variable using the assigned profile memberships, considering that these contain classification errors (Bakk and Vermunt, 2016). Moreover, in order to examine the association between the latent profiles and the dependent categorical variables (e.g., gender), Mplus’ auxiliary (e) function was applied. This approach is based on the Wald chi-square test of statistical significance and uses a pseudo-class method testing the equality of means across profiles (Wang et al., 2005).

## Results

### Latent Profile Analysis of Implicit Theories

In order to identify profiles of parents’ implicit theories of intelligence and self-regulation, latent profile analyses were conducted. Five models with one to five profiles were conducted for model comparisons. Model fit statistics for the optimal number of profiles in the latent profile analysis are displayed in Table 1.

**TABLE 1:** Model fit for the optimal number of profiles in the latent profile analysis.

Number	AIC	BIC	aBIC	LMR	*p*	Entropy
1	1146.987	1170.347	1145.038	–	–	–
2	1125.608	1163.567	1122.441	30.154	0.0182	0.858
3	1089.257	1141.817	1084.873	44.540	0.0066	0.952
4	1087.035	1154.194	1081.432	11.745	0.2290	0.903
5	1070.718	1152.478	1063.898	20.777	0.6242	0.919

*AIC, Akaike’s Information Criterion; BlC, Bayesian Information Criterion; aBlC, sample size adjusted BlC; LMR, Lo-Mendell-Rubin adjusted LRT Test.*

Model fit statistics provided inconsistent results for the optimal number of profiles. AIC and aBIC values were lowest for the five-profile solution, indicating that five profiles were optimal. LMR was not significant for solutions with more than three profiles, suggesting a three-profile solution. Entropy increased from two to three profiles and then declined, suggesting a three-profile solution, too. BIC values were lowest for the three-profile solution, which demonstrated that this was the optimal number of profiles. In sum, most of the model fit statistics provided the three-profile solution. Furthermore, the three-profile solution produced a number of interesting comparisons between profiles and had the clearest interpretation. Therefore, the preferred model is a three-profile solution.

### The Latent Profiles

**FIGURE 1:**
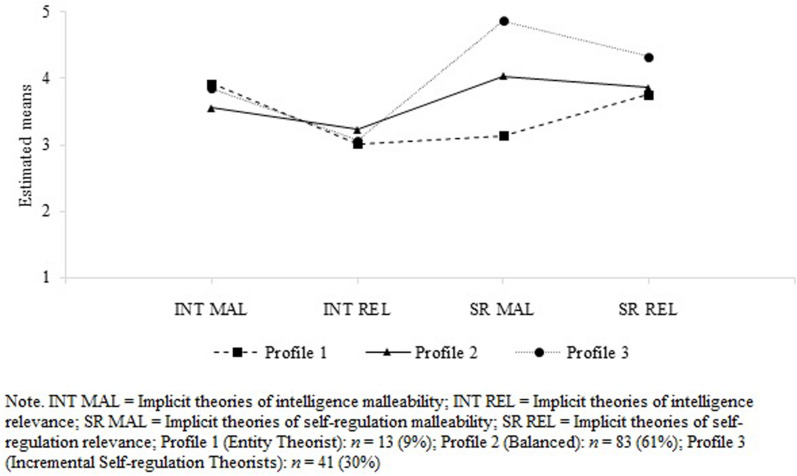
Three-profile solution for the latent profile indicators.

Figure 1 illustrates the three latent profiles and their means on implicit theories on intelligence and self-regulation. The emerged profiles are labeled according to the interpretation of findings as *Entity Theorists*, *Balanced*, and *Incremental Self-regulation Theorists*. As shown in Figure 1, the profiles differ most in their incremental theories of self-regulation.

Parents in profile 1 (9% of the sample, *n* = 13) reported that their child’s intelligence is malleable and moderately relevant for success, while their child’s self-regulation is rather stable and relevant for success. Parents in this group showed the lowest values in their incremental theories of self-regulation and thus exhibited the greatest differences in this variable compared to parents in profiles 2 and 3. We refer to this profile as *Entity Theorists*.

Parents in profile 2 (61% of the sample, *n* = 83) showed similar levels in their incremental theories in both domains as well as in their relevance theories in both domains. They reported that their child’s intelligence and self-regulation are neither particularly stable nor malleable or notably relevant for their child’s success, reflecting balanced levels of both domains of implicit theories. We refer to this group as *Balanced*.

Parents in profile 3 (30% of the sample, *n* = 41) showed the highest values in their incremental and relevance theories of self-regulation. Regarding their incremental and relevance theories of intelligence, this profile showed a similar pattern to profiles 1 and 2. We label this profile as *Incremental Self-regulation Theorists*.

### Differences Between Latent Profiles on Demographic Variables

The data in Table 2 show the means for all of the demographic variables by latent profiles and the full sample. Significance tests for group differences using the pseudo-class method for categorical variables (e.g., gender) and the BCH method for continuous variables (e.g., age) are also reported in Table 2.

**TABLE 2:** Means and standard errors (in parentheses) of demographic variables by latent profiles.

Variable	Full sample		Profile 1^a^		Profile 2^b^		Profile 3^c^		Overall χ*^2^*	Profile 1 vs. 2	Profile 1 vs. 3	Profile 2 vs. 3
**Parent characteristics**												
Gender^1^ (female)	0.87	(0.03)	0.85	(0.10)	0.85	(0.04)	0.93	(0.04)	2.24	0.00	0.54	2.13
Age^2^ (years)	37.42	(0.45)	27.90	(4.53)	30.50	(1.97)	32.92	(2.28)	1.22	0.98	0.27	0.64
Education^1^	0.79	(0.03)	0.47	(0.14)	0.70	(0.13)	0.83	(0.06)	5.75	1.45	5.37*	0.80
Number children^2^	1.93	(0.06)	1.85	(0.22)	1.88	(0.07)	2.07	(0.13)	1.85	0.02	0.82	1.73
**Child characteristics**												
Gender^1^ (female)	0.55	(0.04)	0.53	(0.14)	0.59	(0.06)	0.46	(0.08)	1.74	0.14	0.22	1.86
Age^2^ (years)	4.65	(0.09)	4.40	(0.27)	4.61	(0.12)	4.81	(0.17)	1.80	0.49	1.60	0.87
Self-regulation^2^ (parent-report)	5.34	(0.06)	4.91	(0.18)	5.37	(0.08)	5.47	(0.11)	6.98*	5.39**	6.79*	0.48

Means are based on weighted data. Significance tests for group differences are based on ^1^pseudo-class method or ^2^BCH method with two df for the overall test and one df for the pairwise tests. Statistically significant values are printed in bold; *p < 0.05; **p < 0.01.^*a*^Entity Theorists (9%).^*b*^Balanced (61%).^*c*^Incremental Self-regulation Theorists (30%).

Parents in profile 1 showed the most significant differences from other parents. Parents in this profile had the lowest mean score in parent education compared to parents in the other profiles. This means that 47% of the parents in profile 1 had a university degree, whereas, in profiles 2 and 3, 70% and 83% of the parents were academics, with the differences between profile 1 and profile 3 being statistically significant (χ^2^ = 5.37, *p* = 0.020). Furthermore, parents in profile 1 reported the lowest self-regulatory competence of their child compared to parents in the other profiles, and these differences were statistically significant (profile 1 vs. 2: χ^2^ = 6.79, *p* = 0.009; profile 1 vs. 3: χ^2^ = 5.39, *p* = 0.020). Finally, we found on a descriptive level, that parents in profile 1 were younger, and had fewer and younger children, even though these differences were not statistically significant.

Although the contrasts between profiles 2 and 3 were not statistically significant, almost all parents of profile 3 were mothers (93%), whereas 15% of profiles 1 and 2 were fathers. Moreover, profile 3 had the lowest percentage of daughters (46%) and the highest amount of children (*M* = 2.07, *SE* = 0.13) compared to parents in profiles 1 and 2.

### Differences Between Latent Profiles on Failure Beliefs, Goal Orientation, and Co-regulatory Strategies

The data in Table 3 show the means for failure beliefs, goal orientation, and co-regulatory strategies by profile membership. The first column represents the overall mean for the full sample, and subsequent columns represent the means by latent profiles. In order to explore how the profiles differ by parents’ failure beliefs, goal orientation, and co-regulatory strategies, equality tests of means across profiles using the BCH procedure were conducted. Results of the overall chi-square test as well as the pairwise single-comparisons between groups are reported in the subsequent column of Table 3.

**TABLE 3:** Means and standard errors (in parentheses) of failure beliefs, goal orientation, and co-regulatory strategies by latent profiles.

Variable	Full sample		Profile 1^a^		Profile 2^b^		Profile 3^c^		Overall χ*^2^*	Profile 1 vs. 2	Profile 1 vs. 3	Profile 2 vs. 3
Failure-is-enhancing mindset	3.88	(0.07)	3.65	(0.25)	3.76	(0.08)	4.19	(0.12)	9.58**	0.16	3.86	8.74**
**Goal orientation**												
Learning goals	4.33	(0.04)	4.22	(0.01)	4.29	(0.05)	4.44	(0.06)	5.21	0.41	3.59	3.55
Performance-approach goals	3.03	(0.06)	3.02	(0.15)	3.01	(0.07)	3.06	(0.12)	0.11	0.02	0.04	0.10
Performance-avoidance goals	2.07	(0.05)	2.00	(0.16)	2.16	(0.07)	1.91	(0.09)	4.72	0.87	0.23	4.56*
**Mastery-oriented strategies**												
Praising	4.83	(0.08)	3.95	(1.08)	4.70	(0.09)	5.04	(0.15)	4.42	0.47	0.99	3.73*
Negotiating/discussing	4.17	(0.06)	4.00	(0.19)	4.11	(0.07)	4.37	(0.11)	4.96	0.28	2.86	3.99*
Distraction	3.31	(0.08)	3.85	(0.25)	3.16	(0.10)	3.46	(0.15)	8.07*	6.56*	1.83	2.98
Call for self-regulation	3.09	(0.09)	2.64	(0.15)	2.94	(0.20)	3.24	(0.16)	7.26*	1.40	7.25**	1.40
**Helpless-oriented strategies**												
Giving in	2.39	(0.07)	2.19	(0.21)	2.42	(0.09)	2.40	(0.13)	0.99	0.98	0.69	0.02
Negative pressure	3.57	(0.06)	3.60	(0.13)	3.57	(0.09)	3.57	(0.12)	0.04	0.03	0.00	0.04

Means are based on weighted data. Significance tests for group differences are based on BCH method with two df for the overall test and one df for the pairwise tests. Statistically significant values are printed in bold; *p < 0.05; **p < 0.01.^*a*^Entity Theorists (9%).^*b*^Balanced (61%).^*c*^Incremental Self-regulation Theorists (30%).

The analysis was clearest in distinguishing parents in profile 3 from the other parents. Table 3 shows that parents in this profile reported a failure-is-enhancing mindset significantly more often compared to profile 2 (χ^2^ = 8.74, *p* = 0.003) and pursued performance-avoidance goals less likely than parents in profile 2 (χ^2^ = 4.56, *p* = 0.033). Regarding co-regulatory strategies, parents in profile 3 showed higher values in mastery-oriented strategies. More precisely, parents in profile 3 had higher values in negotiating (χ^2^ = 3.99, *p* = 0.046) compared to parents in profile 2, and significantly higher values in call for self-regulation than parents in profile 1 (χ^2^ = 7.25, *p* = 0.007).

Descriptively, parents in profile 1 had the lowest failure-is-enhancing mindset and learning goal orientation. Furthermore, parents in this profile showed the lowest mean scores for praising, and call for self-regulation as well as the highest value for distraction compared to the other two profiles. As shown in Table 3, the multivariate analysis indicated that at least one of these differences between profiles were statistically significant (χ^2^ = 6.56, *p* = 0.010). On a descriptive level, we also found that parents in profile 1 reported to give in and negotiate least compared to parents in the other profiles even though this difference was not significant.

Profile 2 is characterized by higher values in performance-avoidance goals, which significantly differ from parents in profile 3 (χ^2^ = 4.56, *p* = 0.033). They showed the lowest mean score in distraction compared to the other two profiles, with the differences between this profile and profile 2 being statistically significant in the multivariate analysis (χ^2^ = 6.56, *p* = 0.010).

## Discussion

The present study examined parents’ implicit theories of intelligence and implicit theories of self-regulation simultaneously from a person-centered perspective. We expected that different belief profiles exist and analyzed how the emergent belief profiles are composed concerning demographic variables. Finally, we assumed that the emergent belief profiles differ concerning parents’ attitudes (i.e., goal orientation, failure beliefs) and co-regulatory strategies (i.e., mastery- and helpless-oriented strategies).

### Belief Profiles

The results of the LPA showed that three profiles of implicit theories exist and that most parents (61%) engage in balanced levels of the both examined domains of implicit theories (profile 2). The minority of parents (9%) displayed an entity theory (profile 1), while about one-third of the parents (30%) reported high incremental self-regulation theories (profile 3). The profiles overlap a good deal with the groups observed by Hertel et al. (2019) who studied implicit theories of intelligence and self-regulated learning in students. The groups of Hertel et al. (2019) only differ from the results of the current study in the composition of the group sizes that may result from different research contexts.

The results of the present study support the hypothesis that implicit theories of different domains can co-occur within persons. Although 60% of the parents reported both domains (i.e., intelligence and self-regulation) to be more or less equally malleable and relevant for success (profile 2), 40% of the parents differed in their beliefs across domains. Parents in profile 1 hold an incremental theory in the domain of intelligence while holding rather an entity theory in the domain of self-regulation. Parents in profile 3 perceived the malleability and relevance of their child’s self-regulation to be much higher compared to the domain of intelligence.

Overall, most parents across profiles believed that intelligence and self-regulation are rather malleable and relevant for success, reflecting a ceiling effect. Nevertheless, the greatest differences between profiles became visible in parents’ incremental theories of self-regulation. Compare, for example, profiles 1 and 3. Although both groups were nearly identical in their implicit theories of intelligence, their implicit theories of self-regulation diverge. One explanation might be that parents of preschoolers get to observe and experience situations more often in which their child’s self-regulation becomes more obvious (e.g., respond to external demands, face prohibitions, deal with failure; see Pauen et al., 2019) than their child’s intelligence (that might become more evident later in school life). In early childhood, self-regulatory competencies are developing (Kopp, 1982; Posner and Rothbart, 2000) and parents recognize interindividual differences in children (Bechtel et al., 2016; Pauen et al., 2019). These individual experiences and observations might result in the observed interindividual differences in parents’ incremental theories of self-regulation. Thus, this finding highlights the importance of considering implicit theories of self-regulation beyond the more general implicit theories of intelligence.

Based on the demographic statistics, parents with entity theories (profile 1) were significantly less educated and rated their child’s self-regulatory abilities as lower than parents with high incremental theories (profile 3). These results are in line with research using variable-centered methods (Pomerantz and Dong, 2006; Muenks et al., 2015; Haimovitz and Dweck, 2016; Jiang et al., 2019) finding associations between parents’ implicit theories, education and children’s competencies. Our findings suggest that interventions targeting parents’ implicit theories might especially address low educated parents. As parents’ educational attainment is a significant predictor of children’s self-regulatory abilities (for a meta-analysis see Lawson et al., 2018), interventions are substantial to promote child self-regulation and to buffer the potential negative effect of low educational attainment. However, the associations between profile membership and children’s self-regulatory abilities are possible in both directions (i.e., profile membership predicting child self-regulation and vice versa). For example, parents with entity theories view their child’s self-regulation as stable, show less support for their child, which may result in lower self-regulatory abilities. Otherwise, parents with low self-regulated children may observe less progress and therefore believe that self-regulation is stable. In contrast, parents with high self-regulated children have observed child development and, therefore, think that self-regulation is malleable. As this study is limited to cross-sectional data, we cannot draw any conclusions on the directions of effect. Therefore, these mechanisms have to be addressed in further research.

### Relations Between Latent Profiles and Parents’ Attitudes and Co-regulatory Strategies

The third research question aimed to examine whether the latent belief profiles were associated with parents’ attitudes and co-regulatory strategies. Our findings suggest that parents in different profiles show differentially adaptive or maladaptive patterns concerning their attitudes and co-regulatory strategies. Parents in profile 3 showed the most adaptive attitudes and behaviors compared to the others. They reported to hold more failure-is-enhancing mindsets and to engage in less performance-avoidance goals. These findings are in line with research using variable-centered methods (Burnette et al., 2013; Haimovitz and Dweck, 2016). Regarding co-regulatory strategies, our results add to Moorman and Pomerantz’s (2010) findings that parents with high incremental theories (profile 3) report not less helpless-oriented strategies but more mastery-oriented strategies such as praising, negotiating, and call for self-regulation compared to the other profiles. The only exception emerged for distraction with parents in profile 1 showing higher values than parents in profile 3. As distraction can be both adaptive (Manimala et al., 2000; Stern et al., 2018) as well as maladaptive (Dahlquist and Pendley, 2005) in different situations, the context seems to be a relevant factor. As distraction was measured in a more context-general way in this study, future research should examine parents’ distraction strategies in specific situations. Besides, the relation between profile membership and distraction strategies might also be related to children’s self-regulatory abilities and failure beliefs: Parents who believe that self-regulation is stable engage in distraction strategies in order to avoid frustration and failure since the child cannot self-regulate due to low self-regulatory abilities (see profile 1). Thus, these parents believe that failure is debilitating because failure cannot enhance stable abilities. One may argue that this pattern can be an adaptive response when abilities are low and stable because parents do not overstrain their child. Actually, ample evidence indicates that self-regulatory abilities are malleable (Kopp, 1982; Huizinga et al., 2006; Bernier et al., 2010) and can be enhanced by training and interventions (Kaminski et al., 2008; Diamond and Lee, 2011; Walk et al., 2018; Diamond et al., 2019).

Although there is empirical evidence that parents’ incremental theories of intelligence are negatively associated with controlling and performance-oriented behaviors (Moorman and Pomerantz, 2010), our results show that holding an incremental theory in one domain is not the only important predictor. The positive effects of parents’ incremental theories of intelligence might be less strong when parents hold an entity theory in the domain of self-regulation at the same time (see profile 1). This finding supports the assumption that implicit theories of self-regulation are stronger predictors for domain-related attitudes and behavior than more general implicit theories of intelligence. Here, parents’ implicit theories of self-regulation counteracted the effects of the domain of intelligence.

### Limitations and Further Research

Our study should be interpreted in the light of their limitations. First, we used data from one single sample of preschoolers’ parents and did not replicate the emerging profiles in a second, larger sample, which raises the question of generalization. Anyhow, our three-profile solution is supported by studies examining implicit theories in students (Hertel et al., 2019). Nevertheless, future research should study implicit theories in other samples of parents and examine whether the profiles are the same as in our study. Moreover, even though we did not find any age differences in our sample of three to six years old children, it would be interesting to examine the relations in other age groups, for example, in parents of toddlers or school-aged children. Here, more research is needed.

Second, one might be concerned about the recruitment of the sample via the Internet because we finally could not validate participants’ status as parents. However, most of the participants were recruited via announcements in kindergartens. Thus, we may assume that only parents participated. Nonetheless, we cannot rule out a selection bias of the sample because the caption of the study was related to the role of self-regulation in early childhood. The study might especially have addressed parents who believe that self-regulation is malleable and highly relevant, explaining the high ceiling effect of implicit incremental theories of self-regulation. Furthermore, the sample shows a high proportion of mothers and high-educated parents. In future studies, other cultural contexts and a higher proportion of fathers should be considered. A validation of the emerging profiles in other cultural contexts might be an important next step in further research. For example, cross-cultural studies with Chinese and Finnish students illustrate both similarities and differences in students’ implicit theories with regard to academic achievement (Zhang et al., 2019, 2020). As this study was conducted with a German sample, the question arises if different profiles would emerge when other cultural contexts would be considered: Cross-cultural studies with parents show that Chinese parents seem to emphasize good grades and competition in comparison to Western parents who place a high value on individual growth (Tobin et al., 1989; Sang, 2017). Therefore, considering different cultural contexts might have important implications for parents’ belief profiles.

Third, our study is a cross-sectional study that does not allow any causal interpretation of findings. Future research could use an experimental design where implicit theories of multiple domains can be manipulated, and their effects on parents’ attitudes and behavior can be examined. Besides, future research could examine if the profiles are stable or if parents change profile membership over time. Here, it would be interesting to analyze factors that predict changes in profile membership as well as associated changes in parents’ attitudes and behavior, for example by using analytical techniques such as latent transition analysis.

Finally, we relied on self-reports of all study variables which may increase the risk of common-method variance (Podsakoff et al., 2012) and may be associated with problems of social desirability explaining the null effects for helpless-oriented strategies. We took several steps to reduce social desirability. Data were collected anonymously, participants were asked to fill out seriousness checks, and those who reported not having answered seriously and conscientiously were excluded from the analyses. Additionally, we included a questionnaire testing social desirability, thus ruling out that no social desirability bias as well as no significant correlations with parents’ implicit theories were found. However, future studies should also include observational methods to assess parent-child-interactions.

## Conclusion

Our study showed that implicit theories of intelligence and self-regulation occur in different configurations within parents, with 60% of the parents holding a balanced profile. These differences in belief profiles of parents were also associated with differences in their attitudes and co-regulatory strategies. Incremental self-regulation theorists emerged as the most adaptive configuration for parents’ attitudes and strategies, whereas entity theorists showed rather maladaptive patterns. Our results emphasize the crucial role of implicit theories of self-regulation. This knowledge can be used for interventions targeting parents’ implicit theories. By illustrating that children’s self-regulation is malleable and relevant for success, adaptive configuration for parents’ attitudes and strategies can be promoted. This might in turn impact children’s implicit theories, learning, and development (Blackwell et al., 2007).

## Data Availability Statement

The raw data supporting the conclusions of this article will be made available by the authors, without undue reservation, to any qualified researcher.

## Ethics Statement

The studies involving human participants were reviewed and approved by the Ethics Commission of the Faculty of Behavioral and Cultural Studies, Heidelberg University. The participants provided their written informed consent to participate in this study.

## Author Contributions

MS and SH conceptualized the study. MS collected the data, analyzed them, and wrote the first draft. SH supervised the project. Both authors contributed to the article and approved the submitted version.

## Conflict of Interest

The authors declare that the research was conducted in the absence of any commercial or financial relationships that could be construed as a potential conflict of interest.
